# Palliative care provision for people living with heart failure: The Geneva model

**DOI:** 10.3389/fcvm.2022.933977

**Published:** 2022-08-25

**Authors:** Lisa Hentsch, Piotr Z. Sobanski, Monica Escher, Sophie Pautex, Philippe Meyer

**Affiliations:** ^1^Division of Palliative Medicine, Geneva University Hospitals, Geneva, Switzerland; ^2^Palliative Care Unit and Competence Center, Department of Internal Disease, Schwyz Hospital, Schwyz, Switzerland; ^3^Division of Cardiology, Geneva University Hospitals, Geneva, Switzerland

**Keywords:** palliative care, heart failure, left ventricular assist device (LVAD), quality of life, symptom management

## Abstract

As life expectancy rises and the survival rate after acute cardiovascular events improves, the number of people living and dying with chronic heart failure is increasing. People suffering from chronic ischemic and non-ischemic heart disease may experience a significant limitation of their quality of life which can be addressed by palliative care. Although international guidelines recommend the implementation of integrated palliative care for patients with heart failure, models of care are scarce and are often limited to patients at the end of life. In this paper, we describe the implementation of a model designed to improve the early integration of palliative care for patients with heart failure. This model has enabled patients to access palliative care when they normally would not have and given them the opportunity to plan their care in line with their values and preferences. However, the effectiveness of this interdisciplinary model of care on patients' quality of life and symptom burden still requires evaluation.

## Introduction

As life expectancy increases and the survival rate after acute cardiovascular events improves, the number of people living and dying with serious health-related suffering is growing and this tendency is expected to increase, especially in people aged 70 and over (1). People suffering from chronic heart diseases, whether ischemic or non-ischemic, have been shown to experience a substantial symptom burden and a decrease in quality of life (QoL) that is equal to or even more pronounced than that of patients suffering from cancer (2).

Palliative care (PC) is an approach that aims to improve the QoL of patients with life-limiting diseases, through the thorough assessment and treatment of pain and other symptoms whether physical, psychological, social or spiritual (3). In 2019, the United Nations recognized PC as an essential health care service to be included in the Universal Health Coverage. PC should therefore be integrated in a continuum of care and be available for all people requiring it. Improving QoL has been identified as being as important in public health as prevention, cure and prolonging life (4).

Heart failure (HF) was clearly identified as one of the conditions causing severe health-related suffering that could benefit from PC interventions (4). Many cardiology and PC societies recommend integrating PC in the care of patients living with HF with the aim of improving quality of life (5–9). Some of them clearly specify that this integration should take place regardless of the stage of the disease, which is aligned with the opinion of patients and their relatives who would prefer if PC was offered early in the course of their illness (5, 9, 10). Despite this knowledge, patients living with HF still have limited access to PC, which, despite recommendations, is provided very late in the disease trajectory, for a short period of time, generally in the days preceding death (11). However, this tendency seems to be improving in recent years (12).

Several models for the integration of PC into the care of patients with HF have been suggested, mainly in relation to HF-related hospitalizations (13–15). These models are all designed based on prognosis rather than on the existence of unaddressed needs and therefore only include patients who have a high, short-term, risk of dying. Although early integration of PC is now recommended by both the European Society of Cardiology (ESC) and the European Association for Palliative Care (EAPC) position papers and guidelines for patients with chronic HF, models for the early integration of PC in HF outpatients are surprisingly scarce (9, 16, 17).

At this stage, many questions remain regarding the most effective way to implement PC provision for people living with HF, such as how to identify patients who could benefit from it, when to begin implementing PC into their usual care and how to best assess their needs (18, 19).

Access to PC is mainly dependent on policy, funding, center-based expertise and local resources (20). Models of integration of PC for patients with HF need to be adapted in order to be appropriate to the setting, population and health care system in which it is provided. This paper presents the concept of an integrated PC service for patients with HF in a tertiary HF center based at the Geneva University Hospitals in Switzerland.

## Context

The Geneva University Hospitals (HUG) include eight public hospitals in the canton of Geneva and two clinics, making it the biggest university hospital conglomerate in Switzerland with a total of 13,557 employees for 2,109 beds (21). In 2020, 280,000 patients were hospitalized at the HUG and a total of 1,074,645 out-patient consultations were conducted (21). The HUG are recognized on a national and international level for their expertise in several disciplines including cardiovascular diseases, and collaborate actively with the World Health Organization (21).

In 2017, the Division of Cardiology of the HUG created a HF unit which is mainly dedicated to the ambulatory care of patients with advanced HF, of patients with left ventricular assist devices (LVAD) and of transplant patients. The main objective of the unit is to offer specialized care to these patients in order to avoid unnecessary hospitalizations and increase survival rate, to evaluate the need for and discuss advanced HF therapies and to improve patients' quality of life. In 2021, 4,005 consultations (2,267 nurse-led consultations and 1,738 medical consultations) were conducted in approximately 400 HF or transplant patients.

The HUG provides patients with a network of specialized PC. The Division of Palliative Medicine includes three acute PC units with a total of 36 beds, two mobile PC teams, one working in the acute care and rehabilitation hospitals and one in the geriatric and psychiatric hospitals. A mobile PC team provides care to patients living in the community, either at home or in nursing homes. An outpatient PC consultation opened in 2019 where an average of 202 patients per year have been assessed and followed. In 2021, 2,626 patients, aged 16 to 103, have benefited from specialized PC at the HUG.

## Implementing palliative care for patients with heart failure

In 2018, we initiated an interdisciplinary working group including a PC physician, a PC nurse, a cardiologist and three cardiology nurses. The aim of this group was to improve PC provision for patients suffering from HF by implement PC interventions at all stages of the patient's trajectory. Based on national and international recommendations, we define three PC interventions, that could easily be implemented (9, 16, 22, 23):

1. Creating an interdisciplinary consultation for patients with advanced HF.
2. Implementing a routine PC consultation for all patients undergoing evaluation for a LVAD or a heart transplant.
3. Training sessions for the cardiology team in order to provide them with the skills necessary to perform symptom evaluation and initiate advance care planning.

The idea underpinning each of these interventions was to provide early integrated PC for patients suffering from HF, since we had acknowledged the fact that PC was often implemented too late in the disease.

An important aspect of our care model was to ensure that patients were still followed primarily by their cardiologist, as continuity of care is a core component of good quality care and of patient satisfaction with care (10).

### Interdisciplinary consultations

Patients are initially identified by HF physicians or nurses, based on a screening of PC needs, concerning mainly symptom burden, complex psycho-social circumstances or the identification of potentially divergent goals of care between patient and healthcare professionals. This evaluation is currently based on a narrative review as no validated tool currently exists in French for the evaluation of PC needs in patients with HF.

After a patient has been identified, an initial consultation is conducted by a PC physician and a cardiologist either together or back-to-back depending on the physicians' availability. This consultation is aimed at presenting the scope of PC, conducting a thorough evaluation of symptoms and psycho-social concerns and evaluating the patient's knowledge of disease, values and willingness to discuss advance care planning. If the primary consultation cannot be conducted by the cardiology and the PC physicians simultaneously, one of the cardiology nurses will generally accompany the patient to the first PC consultation in order to complement the information provided by the PC specialist and later communicate important elements of the discussion to the cardiology team. This consultation is then transcribed in a report that is included in the patient's electronic medical record and forwarded to the patient's cardiologist, general practitioner, home care service and any other professionals involved in the patient's care.

After the first consultation, patients are offered PC follow-up consultations, conducted by a PC physician, at a frequency adapted to suit the patient's needs and symptoms. During these follow up consultations, all patients are assessed for symptom burden, psychological, social and spiritual needs and offered the possibility of discussing advance care planning and document its conclusions (e.g., in the form of advance directives). Referral to a social assistant or home care services can be organized if required. Psychological support is provided by psychologists and psychiatrists from the ambulatory psychiatry division of the HUG. Spiritual issues, frequently independent or beyond religious concerns, are generally assessed during the first PC consultation. Spiritual assistance can be provided by one of the chaplains working with the HUG, if they do not already have support from their community chaplain. Symptoms are assessed by the Edmonton Symptom Assessment System (ESAS) (24). This validated tool allows patients to rate intensity of nine common symptoms in PC, such as pain, breathlessness, anxiety and drowsiness on a scale of 0 (none) to 10 (severest). If breathlessness is reported, patients are asked to complete the Dyspnea-12 questionnaire. This tool was developed and validated in many cardiopulmonary diseases, including HF, for breathlessness assessment based on physical and affective components (25–27). This evaluation helps inform the need for non-pharmacological (e.g., cardiovascular rehabilitation) and/or pharmacological (e.g., opioids) interventions to alleviate breathlessness and complementary approaches such as hypnosis or sophrology to improve general well-being and crisis management. Social needs are assessed through discussion and patients are referred to appropriate services as required. All patients are offered the possibility to rediscuss advance care planning throughout the PC follow-up triggered by the progression of symptoms and needs and encouraged to name a healthcare surrogate. Follow-up consultations mainly focus on the patient's needs, but always include a comprehensive symptom assessment.

### Pre-LVAD and -transplant palliative care consultation

Heart transplantation remains the best treatment option offered to patients with advanced HF. However, only a small number of eligible patients undergo heart transplantation and alternative options should be discussed early on in the process.

As of 2020, all patients electively hospitalized for a five-day pre-transplant or pre-LVAD workup, either as destination therapy or as a bridge to transplant, were systematically scheduled for a PC consultation. The PC consultation is planned in the patient's agenda, alongside other consultations such as radiological exams, pulmonary evaluation and psychological assessment. The consultation is conducted by a senior PC physician. The main topics addressed are the aims of PC and advance care planning. Patient's lived experience of the disease as well as hopes and fears regarding the planned intervention, its results and the life thereafter are discussed, and information about advance directives and a healthcare surrogate are given. Depending on the patient's needs, follow-up ambulatory PC consultations can be offered. Since October 2020, 11 patients have undergone a PC evaluation before a LVAD implantation or heart transplant. No patient refused the PC consultation. One patient left the hospital early and did not complete the work-up, including PC assessment.

### Training sessions

Healthcare providers' lack of knowledge about PC has been found to be one of the main barriers to providing quality PC to patients with HF (28). The PC team therefore started by providing knowledge about the definition and aim of PC through a series of presentations/coaching sessions given to the interprofessional HF team. A PC specialist nurse then conducted individual training sessions with the cardiology nurses on how to evaluate symptom burden and initiate advance care planning with patients.

### The Geneva model

**Figure 1:**
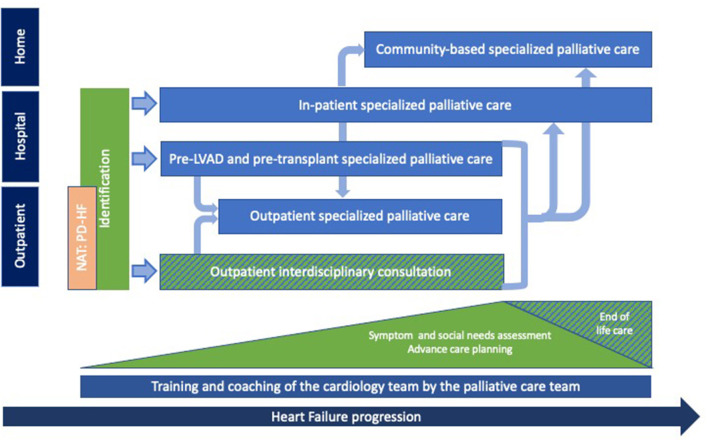
Palliative care provision for patients with heart failure: The Geneva model. PC provided by the palliative care team (light blue) for patients with HF according to setting and disease progression. Identification of eligible patients and provision of general palliative care, mainly symptom assessment, social needs evaluation and discussions about advance care planning, are provided throughout the disease trajectory by the interprofessional cardiology team (green). End of life care is provided by the cardiology and the PC team. Training and coaching sessions of the interprofessional cardiology team are conducted continuously by the PC team (dark blue). The NAT: PD-HF (orange) is currently being integrated to our model and should allow for an earlier access to PC provision for patients with HF. PC, palliative care; HF, heart failure.

International recommendations suggest integrating PC at every stage of the HF trajectory, in a flexible, adaptable way (9, 16). Based on the development of the previously listed interventions, we designed a model of care for the integration of PC for patients with HF according to care setting (e.g., in hospital, in out-patient consultations or in the community) and disease progression (Figure 1). Integration of specialized PC is done after identification of PC triggers by cardiologists/HF nurses in the outpatient or in-patient setting. Depending on the patient's situation, specialized PC is then either introduced during hospitalization for acute HF, at a pre-LVAD or pre-transplantation consultation or during an interdisciplinary outpatient consultation. After this first contact, specialized PC can continue to be provided, according to the setting, as an interdisciplinary consultation, as a specialized PC outpatient consultation, as a specialized PC in-patient consultation or as a home-based specialized PC consultation.

Certain elements of general PC are now being carried out by the cardiology team such as assessing symptoms, social needs and initiating advance care planning discussions. The palliative care team simultaneously provides support by providing members of the cardiology team with presentations on palliative symptom assessment/management and coaching sessions on how to initiate advance care discussions with patients and their families. With the aim of maintaining continuity of care, end of life care is provided by the cardiology and PC team, as required.

Since the beginning of this collaboration in October 2020, the number of PC consultations for patients with HF has been increasing in all settings, whether for ambulatory or pre-transplant patients (Table 1). In the out-patient setting, patients benefited from an average 1.5 consultations (range-1–4) that lasted from 30 min to an hour and a half, depending on the patient's needs. All of them were offered the possibility of discussing advance care planning. More than half of all patients completed advance directives following the PC consultation.

**Table 1:** Numbers of HF patients having benefited from a consultation with a PC physician per year since October 2020, outside specialized PC units.

**Year**	**Inhospital**	**Ambulatory**	**Pre-transplant**
2020	25	0	3
2021	40	5	6
2022 (January–March)	7	9	3

## Discussion

Chronic ischemic and non-ischemic heart diseases are amongst the leading health conditions driving the global burden of serious health-related suffering in the world, and the number of affected individuals is expected to increase in the near future (1). Finding ways to support people throughout a chronic disease trajectory even when improvement of the underlying condition can be expected, as in patients awaiting transplantation, should be a health priority. Despite the fact that integration of PC for patients with HF was shown to improve QoL and patient satisfaction, it is still underprovided (29).

In 2017, Rogers et al. evaluated an interdisciplinary PC intervention in addition to evidence-based HF care (14). This intervention showed a significant benefit to the patients' QoL, anxiety, depression and spiritual well-being compared to usual care alone (14). The core components of the intervention which were symptom management and identifying goals of care were the same as in our intervention. However, the study only included patients with a high risk of 6-month mortality, therefore late in the disease.

A recent systematic review and meta-analysis conducted on PC interventions for patients with HF identified 15 studies, only two of which involved outpatients (29). Rabow et al. (30) compared HF patients followed by a general medicine outpatient clinic (usual care) with HF patients benefiting from usual care and PC team consultations. This study showed improvement in patient outcomes such as dyspnea, anxiety and spiritual well-being. In the second study, Evangelista et al. examined the effects of an outpatient PC consultation on symptom burden and QoL in patients with symptomatic HF (17). In this study, patients were recruited in an inpatient setting during an acute HF exacerbation and given an appointment for a PC consultation on the same day as their next outpatient cardiology consultation. Outcomes were evaluated 3 months after that single PC consultation and showed an improvement of symptom burden, depression and QoL compared to the usual care group. Although this study involved the PC and the HF teams, consultations were conducted separately and there were no elements of collaboration between the two teams. It can be hypothesized that a close collaboration between the PC and the HF team may further improve patient-reported outcomes by the early implementation of PC in the disease trajectory and the early collaborative discussions concerning goals of care. This may also, as in cancer patients, reduce the number of futile or harmful interventions at the end of life (31, 32).

Although many models of care for the integration of PC for patients with HF have been suggested it seems important to adapt these models to the local setting, resources and needs (29). Furthermore, it seems important to work together and offer interdisciplinary support in a collaborative manner as opposed to separate PC consultations (30). Evidence shows that patients with HF may have unfavorable impressions of PC (33). Patients value continuity of care and trust their cardiologist, who has often followed them for many years, to provide them with the best, most appropriate care. The integration of PC as an element of comprehensive cardiological care, as provided in our model, assures that the interprofessional cardiological team remains in charge of HF patients' care throughout the disease trajectory.

One of the aims of our interdisciplinary collaboration was to provide healthcare professionals working at the HF unit with the knowledge and tools to identify patients who could benefit from PC. This is a unique feature of our model that focuses on empowering healthcare professionals who may not be initially at ease with PC in providing what is now recognized as good practice for patients with HF (34). This element may be particularly relevant in settings that may have limited resources in specialist PC. As the number of patients in need of PC is constantly increasing, there is an urgent requirement for non-PC specialists to be able to provide general PC. Currently, our model has focused on training nurses and physicians. Other members of the interprofessional cardiology team such as healthcare workers and physiotherapists working with patients during cardiovascular rehabilitation should also be involved in the collaboration so as to create a global culture of care centered on maintaining HF patients' QoL.

Members of the PC teams have also benefited from this interdisciplinary collaboration. Indeed, it has provided the PC team with important knowledge on the needs and trajectory of patients with non-oncological diseases such as HF. It has also offered valuable insight on the management of this population's expectations, in particular regarding the prospect of life-prolonging therapies (e.g., LVAD implantation) in the context of a life-limiting disease.

One of the remaining questions is when and who should receive PC (14, 19, 33). As with oncology patients, it has been suggested that HF patients be referred based on a high risk of dying, poorly controlled symptoms and psychosocial-spiritual distress, hospitalization and discharge or end-of-life transition (18). However, this approach often led to PC being offered very late in the disease course and being reactive as opposed to pro-active. Referring HF patients to PC when they are unwell, does not leave much place for anticipation and contributes to the erroneous perception that PC is only provided to people whose health is deteriorating and prognosis obviously bad. Changing the pattern of care from referral to PC after exhaustion of cardiological treatments, to involving PC into the care, as the needs emerge, eliminates the main barriers among health care providers (who do not need to give up their patients to the PC team) and patients (who do not need to be considered as imminently dying) and allows to focus on QoL during the entire disease journey and not only during the dying phase.

There are currently no French-validated tools for the identification of HF patients who could benefit from PC. The Needs Assessment Tool: Progressive Disease - Heart Failure (NAT: PD-HF) was first developed nearly 10 years ago to identify and inform the management of physical and psycho-social issues experienced by patients suffering from chronic HF (35). It is a tool that is completed in <5 min and can guide physicians when assessing the PC needs of patients with HF and their relatives and help to identify those requiring specialist PC care. The German translation and validation has recently been published by our colleagues in the German-speaking region of Switzerland and we are now collaborating with them on the validation of the French translation (36). We believe implementing the NAT: PD-HF may help increase the number of HF patients receiving PC, based on their needs.

## Future prospects

The idea behind our concept is to provide person-centered care focused on improving the QoL of people living with cardiovascular diseases, particularly HF, during the entire course of their diseases, based on their needs and independent of treatment options and prognosis. More than a single intervention, we have developed a close collaboration between the cardiology and PC teams. Regular interaction has also brought the opportunity to work on streamlining certain procedures such as designing protocols in anticipation of dealing with the end-of-life and death of patients with implanted LVAD or an implantable cardioverter-defibrillator. As LVAD is a relatively recent therapy, there is little evidence or information about the process and a protocol that included contextual information was needed in order to help the physicians who would have to care for these patients (37–39). Indeed, the inactivation of mechanical circulatory support is emotionally challenging and bears ethical dilemmas for most healthcare professionals, patients and their relatives.

We are currently finalizing a guideline to help healthcare professionals care for patients implemented with a LVAD at the end of their life. This guideline includes a presentation of the HEARTMATE 3^TM^, to whom it is destined, main complications, required maintenance and care (40). The guideline describes the step-by-step procedure to inactivate the LVAD including discussions that should be held with the patient and his caregivers and how to guide the patient and his/her family through the process. A pocket guide has also been conceived for physicians on how to inactivate the LVAD alarms. Once completed, this guideline will have to be reviewed by the healthcare professionals involved in the procedure, mainly cardiologists, cardiology technicians, intensive care and internal medicine specialists and the PC team.

## Limitations

Our model of care was based on needs identified by healthcare professionals, mainly physicians and nurses, working with HF patients. We acknowledge the fact that our model could have benefited from the input of patients and caregivers. This could be done through focus groups in order to refine the process.

We acknowledge the subjectivity underlying the referral of HF patients to the PC team in our model. This is due to the absence of a validated instrument in French to identify HF patients who could benefit from specialized PC. In order to address this, we are actively working on the French validation of the NAT: PD-HF.

Although it was not the aim of this paper, we recognize that our model has yet to be tested for its effectiveness in improving patients' QoL and symptom burden as well as its impact on healthcare resource utilization. We plan to study these different aspects in the near future.

Furthermore, we are aware that this model was constructed following a needs assessment conducted in the specific HF unit of a tertiary high-income country and may not be appliable to other settings. However, the specialized PC team is small with only one physician currently conducting outpatient consultations. As mentioned previously, PC should be provided by every healthcare professional involved in the care provided to HF patients and not only by a specialist PC team. This model does not therefore require a broad range of PC specialists.

We are also aware that the success of any collaboration is dependent on the motivation of all parties involved, especially the discipline leaders. Healthcare professionals working with HF patients have many pre-conceived ideas about PC and often mistake PC for imminent end-of-life care. We are extremely privileged to be working with health care professionals that recognize the potential benefits of a collaboration between the cardiology and PC teams for patients suffering from HF and acknowledge the fact that this may be a limiting factor to the integration of PC for HF patients in many other settings.

## Data availability statement

The original contributions presented in the study are included in the article/supplementary material, further inquiries can be directed to the corresponding author.

## Author contributions

All authors listed have made a substantial, direct, and intellectual contribution to the work and approved it for publication.

## Conflict of interest

The authors declare that the research was conducted in the absence of any commercial or financial relationships that could be construed as a potential conflict of interest.

## Publisher's note

All claims expressed in this article are solely those of the authors and do not necessarily represent those of their affiliated organizations, or those of the publisher, the editors and the reviewers. Any product that may be evaluated in this article, or claim that may be made by its manufacturer, is not guaranteed or endorsed by the publisher.
