# Erratum: Models of palliative care provision for people living with heart failure: The Geneva model

**DOI:** 10.3389/fcvm.2023.1176640

**Published:** 2023-03-21

**Authors:** Frontiers Production Office

**Affiliations:** Frontiers Media SA, Lausanne, Switzerland

**Keywords:** palliative care, heart failure, left ventricular assist device (LVAD), quality of life, symptom management

An Erratum on Palliative care provision for people living with heart failure: The Geneva model By Hentsch L, Sobanski PZ, Escher M, Pautex S and Meyer P. (2022) Front. Cardiovasc. Med. 9:933977. doi: 10.3389/fcvm.2022.933977

An omission to the funding section of the original article was made in error. The following sentence has been added: “Open access funding was provided by the University Of Geneva”.

The original version of this article has been updated.

